# Loss of symbiont infectivity following thermal stress can be a factor limiting recovery from bleaching in cnidarians

**DOI:** 10.1038/s41396-020-00742-8

**Published:** 2020-08-21

**Authors:** Mariko Kishimoto, Andrew H. Baird, Shinichiro Maruyama, Jun Minagawa, Shunichi Takahashi

**Affiliations:** 1 National Institute for Basic Biology, 38 Nishigonaka, Myodaiji, Okazaki, 444-8585 Japan; 2 Department of Basic Biology, School of Life Science, The Graduate University for Advanced Studies, SOKENDAI, Okazaki, Aichi Japan; 3 ARC Centre of Excellence for Coral Reef Studies, James Cook University, Townsville, QLD 4811 Australia; 4 Department of Ecological Developmental Adaptability Life Sciences, Graduate School of Life Sciences, Tohoku University, 6-3 Aramaki-aza-Aoba, Aobaku, Sendai, 980-8578 Japan

**Keywords:** Microbial ecology, Biodiversity

## Abstract

Increases in seawater temperature can cause coral bleaching through loss of symbiotic algae (dinoflagellates of the family Symbiodiniaceae). Corals can recover from bleaching by recruiting algae into host cells from the residual symbiont population or from the external environment. However, the high coral mortality that often follows mass-bleaching events suggests that recovery is often limited in the wild. Here, we examine the effect of pre-exposure to heat stress on the capacity of symbiotic algae to infect cnidarian hosts using the Aiptasia (sea-anemone)-Symbiodiniaceae model system. We found that the symbiont strain *Breviolum* sp. CS-164 (ITS2 type B1), both free-living and in symbiosis, loses the capacity to infect the host following exposure to heat stress. This loss of infectivity is reversible, however, a longer exposure to heat stress increases the time taken for reversal. Under the same experimental conditions, the loss of infectivity was not observed in another strain *Breviolum psygmophilum* CCMP2459 (ITS2 type B2). Our results suggest that recovery from bleaching can be limited by the loss of symbiont infectivity following exposure to heat stress.

Cnidarians including reef-building corals harbor endosymbiotic dinoflagellates of the family Symbiodiniaceae, from which they derive the majority of their energy. Therefore, the breakdown of the symbiotic relationship, a process known as bleaching, can result in the host starving. However, bleaching is not always lethal because symbiont densities can recover [1, 2]. Recovery from bleaching is driven mainly by symbiotic algae that remain within the bleached corals (the residual population) dividing and spreading throughout the colony [3], and also possibly through the recruitment of free-living symbiotic algae from the external environment [4]. In the last few decades, coral cover has drastically decreased in many regions, due to frequent mass coral bleaching events caused by global warming [5], implying that recovery from bleaching is often limited by unknown factors. In the present study, we demonstrate that both free-living and residual symbiont cells lose their capacity to infect cnidarian host cells once they are exposed to high temperature stress, and present this mechanism as a limiting factor for the host’s recovery from bleaching.

We first examined the effect of pre-exposure to high temperature on infectivity using aposymbiotic *Exaiptasia pallida* (or “Aiptasia”) polyps (Supplementary Fig. 1) and cultured strains of *Breviolum* sp. CS-164 (ITS2 type B1). Symbiotic algae and polyps were separately incubated at either 25 or 32 °C for 3 days. Following this initial treatment, polyps were inoculated with symbiotic algae at 25 °C for 3 days. Infectivity was then determined by counting the number of algae in the tentacles where algal colonization occurs quickly and individual symbiont cells are easily visualized [6]. When both symbiotic algae and hosts were pre-exposed to 25 °C, significant numbers of algae were seen in tentacles (Fig. 1a). In contrast, the number (Fig. 1a) and density (Fig. 1b) of algae in the tentacles were significantly lower when both the algae and the host were exposed simultaneously to 32 °C, and when the algae alone were exposed to 32 °C. Thus, symbiotic algae, but not host polyps, lose their capacity to form a symbiotic relationship once they are exposed to high temperature. Neither cell viability measured by Evans blue staining (Supplementary Figs. 2a, b and 3) nor cell density (Supplementary Fig. 2c) differed between CS-164 cells exposed to 25 and 32 °C, indicating that infectivity was not lost by the lethal damage to cells. We repeated this experiment with another strain, *B. psygmophilum* CCMP2459 (ITS2 type B2). In contrast to the results with CS-164, high temperature had no effect on infectivity of CCMP2459 (Supplementary Fig. 4a). These results demonstrate that symbiotic algae can lose their capacity to infect host cells following exposure to high temperature and that thermal sensitivity differs between these two algal strains (Fig. 1b and Supplementary Fig. 4a).

**Fig. 1:**
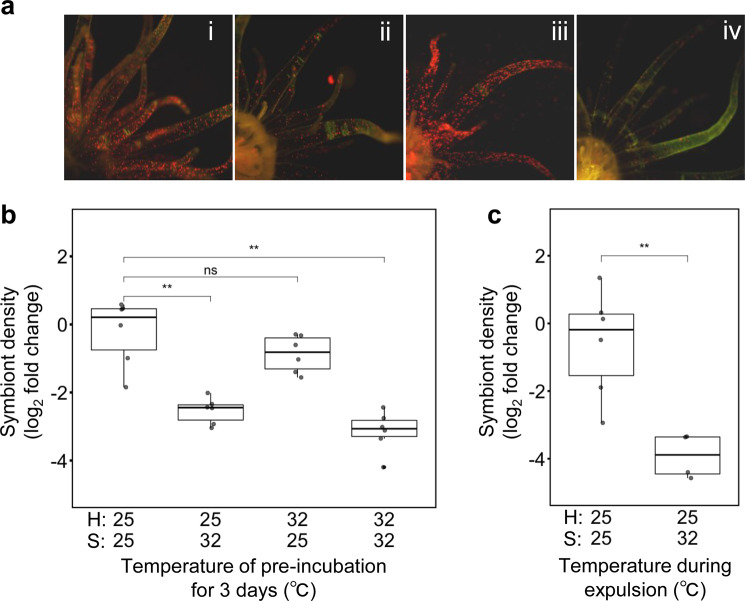
Loss of infectivity in *Breviolum* sp. CS-164 following exposure to elevated temperature. **a** Fluorescent photographs of Aiptasia polyps 3 days after culturing with symbiont cells in four different treatments (i) neither symbionts nor polyps exposed to high temperature (32 °C) for 3 days, (ii) only symbionts exposed to high temperature, (iii) only polyps exposed to high temperature, (iv) both symbionts and polyps exposed to high temperature. Red dots show chlorophyll fluorescence from algal symbionts. **b** The density of symbionts in tentacles was measured 3 days after culturing Aiptasia polyps (H) with symbiont cells (S) in four different treatments, as indicated below the panel and outlined in the text. **c** The density of symbionts was measured 3 days after culturing Aiptasia polyps with symbiotic algae in different treatments. In this experiment, symbiotic algae that had been expelled from Aiptasia polyps cultured at 25 or 32 °C for 3 days were used to infect Aiptasia. **b**, **c** Values are log_2_ fold changes with respect to the samples without any temperature treatment. Each point represents an independent experiment. ns, not significant (with *p* > 0.05); **, *p* < 0.01.

We next examined whether the loss of infectivity following pre-exposure to high temperature also occurs when symbiotic algae are in symbiosis with the host rather than free living (Fig. 1c and Supplementary Fig. 4b). We prepared symbiotic Aiptasia polyps with either CCMP2459 or CS-164 by separately inoculating them in aposymbiotic polyps, and then exposed each group to either 25 or 32 °C for 3 days. Algae expelled from the polyps during this treatment were collected and then used to inoculate aposymbiotic Aiptasia at 25 °C. In CS-164, after 3 days of inoculation, symbiont density in Aiptasia became lower with algae collected at 32 °C than 25 °C (Fig. 1c). However, in CCMP2459, there was no difference in the infectivity between algae collected at 25 and 32 °C (Supplementary Fig. 4b). Our results demonstrate that symbiont cells, both free-living and in symbiosis, can lose infectivity following exposure to high temperature and that thermal sensitivity differs between these two algal strains.

We then tested whether or not the temperature-induced loss of infectivity was reversible (Fig. 2). Free-living CS-164 cells were pre-exposed to 25 or 32 °C for either 2 or 3 days after which they were allowed to recover for a maximum of 10 days at 25 °C. After these treatments, symbiotic algae were used to inoculate aposymbiotic Aiptasia polyps at 25 °C for 3 days. Cells with pre-exposure to 32 °C for 2 days had lower infectivity but regained the capacity to infect host cells after a 5-day recovery period (Fig. 2). However, after 3 days exposure, infectivity gradually recovered but remained lower than the controls even after 10 days (Fig. 2). Our results demonstrate that the loss of infectivity following temperature stress is reversible in algal cells but a longer exposure to heat stress increases the time taken to reverse the loss of infectivity.

**Fig. 2:**
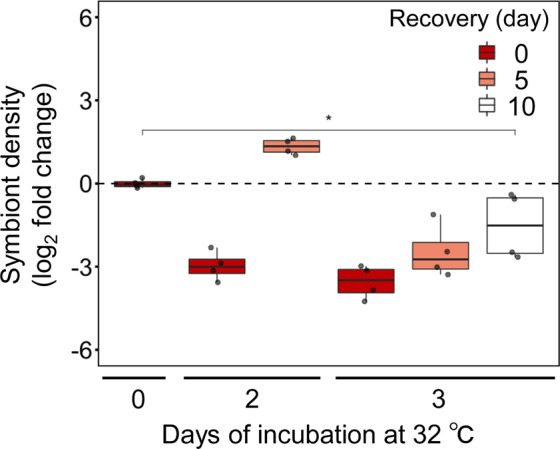
Reversibility of the lost infectivity upon the exposure to elevated temperature in *Breviolum* sp. CS-164. The density of symbionts in tentacles was measured following treatments as described in the text and shown by the relative to control. Values are log_2_ fold changes with respect to the control. The box and line represent the quartiles and median, respectively. Each point represents an independent experiment. *, *p* < 0.05.

In the present study, infectivity was tested by introducing symbiotic algae directly into the host’s body cavity, suggesting that the loss of infectivity seen in our experiments is likely due to a failure of the host to take up the algal cells via phagocytosis or for symbiont cells to persist within host cells. Factors such as symbiont cell size [7] and the symbiont surface glycome [8, 9] are potentially important determinants of symbiont uptake and persistence, though we still know little about this topic (see review [10]). Furthermore, it is unknown how these various discriminatory factors are influenced by thermal stress.

In coral larvae and juveniles, the initiation of symbiosis with algae is reduced at high temperatures, suggesting that global warming will complicate the relationship between host and symbiont [11–13]. However, the mechanism for this reduction in the rate of symbiosis establishment is not clear. Our results suggest that the loss of symbiont infectivity is one possible cause of this phenomenon.

Recovery of symbiont densities following coral bleaching relies on a supply of symbiont cells either from within the host or from the external environment. Our results show that symbiotic algae, both free-living and symbiotic, lose the capacity to infect the host following exposure to high temperature stress (Fig. 1). This loss of infectivity is reversible but dependent on the duration of the thermal stress (Fig. 2). Thus, following coral bleaching events, especially those induced by thermal anomalies that can last for weeks, symbiont densities within the host are unlikely to recover in time to avoid the host starving due to physiological compromise of the symbionts, rather than the host. Nonetheless, given the differences in sensitivity between two strains tested (Fig. 1b and Supplementary Fig. 4a), if heat tolerant symbionts are available in the environment, this might provide a chance for recovery.

## Supplementary information

Supplementary Information
