# Supplementary material for: Loss of symbiont infectivity following thermal stress can be a factor limiting recovery from bleaching in cnidarians
Source: ISME J. 2020 Aug 21;14(12):3149–52. doi: 10.1038/s41396-020-00742-8 (PMC7784860; doi:10.1038/s41396-020-00742-8)
Supplement: Supplementary file 1 — Supplementary Information [file 41396_2020_742_MOESM1_ESM.pdf]

Loss of symbiont infectivity following thermal stress can be a factor limiting recovery from bleaching in cnidarians

Mariko Kishimoto, Andrew H. Baird, Shinichiro Maruyama, Jun Minagawa, Shunichi Takahashi

This PDF file includes:

- Materials and Methods
- Supplementary Figures 1 to 4

Materials and Methods

Cultures and growth conditions

Sea anemone *Exaiptasia pallida* (strain H2, commonly called Aiptasia) polyps were provided by Professor John Pringle (Stanford University). To eliminate symbionts, they were incubated at 34 °C in the dark for 1 month. The elimination of all symbionts was confirmed by the inability to detect any fluorescence from symbiotic algae under a fluorescence microscope (Leica M165FC) under 200 X magnification. The aposymbiotic Aiptasia polyps were cultured in filtered artificial seawater (REI-SEA marine 2; IWAKI) at 25 °C in darkness and fed freshly hatched *Artemia* nauplii once a week, until used in the experiments.

Symbiodiniaceae CCMP2459 (ITS2 type B2) was obtained from the National Center for Marine Algae and Microbiota (USA), and Symbiodiniaceae CS-164 (ITS2 type B1) from the Australian National Algae Culture Collection (AUS). To ensure that cultures were monoclonal, obtained cultures were subcultured from a single cell and their genotypes confirmed in a previous study [1]. Symbiont cells were grown in filtered (0.22 μm pore filter; Steritop-GP Filter Unit, Merck Millipore) artificial seawater (sea salt; Sigma-Aldrich) containing Daigo's IMK medium for marine microalgae (Wako). They were cultured in a growth cabinet MLR-350 (SANYO) at 25 °C under a cycle of 12 h of fluorescent light (100 μmol photons m^{-2} s^{-1}) and 12 h of darkness.

Aiptasia infected with symbionts were obtained by culturing aposymbiotic Aiptasia polyps with cultured symbiont cells (40,000 cells ml^{-1}) in a 10 cm diameter dish with 50 ml of artificial seawater. Polyps were cultured at 25 °C under a cycle of 12 h of fluorescent light (100 μmol photons m^{-2} s^{-1}) and 12 h of darkness. Infection of symbiont cells into the host was confirmed by the change in coloration (white/brown). They were fed freshly hatched *Artemia* nauplii once a week, until used.

Temperature shock treatment

Cultured symbiotic algae were collected by centrifugation (1,500 g for 3 min) and seawater was replaced with new seawater (REI-SEA marine 2; IWAKI). Aposymbiotic Aiptasia polyps were

placed in 6-well plates with 5 ml of fresh seawater. For temperature treatments, symbiont cells and Aiptasia polyps were separately incubated at a growth temperature of 25 °C or an elevated temperature of 32 °C in the dark. To shock symbiont cells within Aiptasia polyps, symbiotic polyps were incubated at 25 °C or 32 °C in the dark for three days. Symbiont cells expelled from polyps were collected by centrifugation (1,500 g, 3 min) and re-suspended in fresh seawater for the use in experiments.

Recovery of infectivity

Following temperature shock treatments, Symbiodiniaceae cells were placed at 25 °C under a cycle of 12 h of fluorescent light (100 µmol photons m^{-2} s^{-1}) and 12 h of darkness in seawater (REI-SEA marine 2; IWAKI) for a maximum of 10 days. Then, cells were used for infection test.

Quantification of infectivity

An Aiptasia polyp was individually placed in a 6 well-plate with 5 ml of artificial seawater. Symbiont cells (approximately 9000 cells) were collected by centrifugation (1,500 g for 3 min) and mixed with fresh *Artemia* nauplii (3-5 individuals in 3 µl seawater) for making a pellet. *Artemia* nauplii were used to accelerate the introduction of an algal pellet into the stomach cavity. The pellet was directly fed to Aiptasia using a glass Pasteur pipet. Aiptasia polyps were then incubated at 25 °C for 3 days under a cycle of 12 h of light (100 µmol photons m^{-2} s^{-1}) and 12 h of darkness for infection. Algal density (algal number per area) in Aiptasia was measured by counting algal cells in tentacles with a stereo fluorescence microscope (Leica M165FC or M205FA) after the incubation.

Analysis of algal cell viability and density

The viability of algal cells was measured by Evans blue staining following a previous study [2]. Evans blue stains cells with compromised membrane structures. We first examined with a light

microscope (Olympus CX43) that CS-164 cells are stained by Evans blue after the expose to heat shock at 80 °C for 1 hour (Supplementary Fig. 3). Less chlorophyll fluorescence was observed using an inverted fluorescence microscope (Olympus IX71) with filters (WIG filter: Excitation 520~550nm band pass, Detection 580nm~) in Evans blue-stained cells, indicating that Evans blue staining is available for the detection of damaged CS-164 cells.

Symbiont cells (40,000 cell ml^{-1}; 1 ml) were incubated at 25 °C or 32 °C in the dark for 3 days. Then, cells were used for the Evans blue staining [2]. Approximately, 50 cells were used for each test. Observations were taken under a light microscope (CX43 Olympus) and a fluorescence microscope (Olympus IX71).

For monitoring cell density, algal cells (40,000 cell ml^{-1}; 10 ml) were incubated at 25 °C or 32 °C in the dark for 5 days. Algal cell density was measured by counting cells using a hemocytometer under a microscope.

Statistics and reproducibility

All statistical tests were performed using R 3.5.2 software. Statistical analysis between two samples was performed using Welch's *t*-test. $P < 0.05$ was considered statistically significant.

Supplementary references

1. Biquand E, Okubo N, Aihara Y, Rolland V, Hayward DC, Hatta M, et al. Acceptable symbiont cell size differs among cnidarian species and may limit symbiont diversity. *ISME J* 2017; **11**: 1702–1712.

2. Morera C, Villanueva MA. Heat treatment and viability assessment by Evans blue in cultured *Symbiodinium kawagutii* cells. *World J Microbiol Biotechnol* 2009; **25**: 1125–1128.

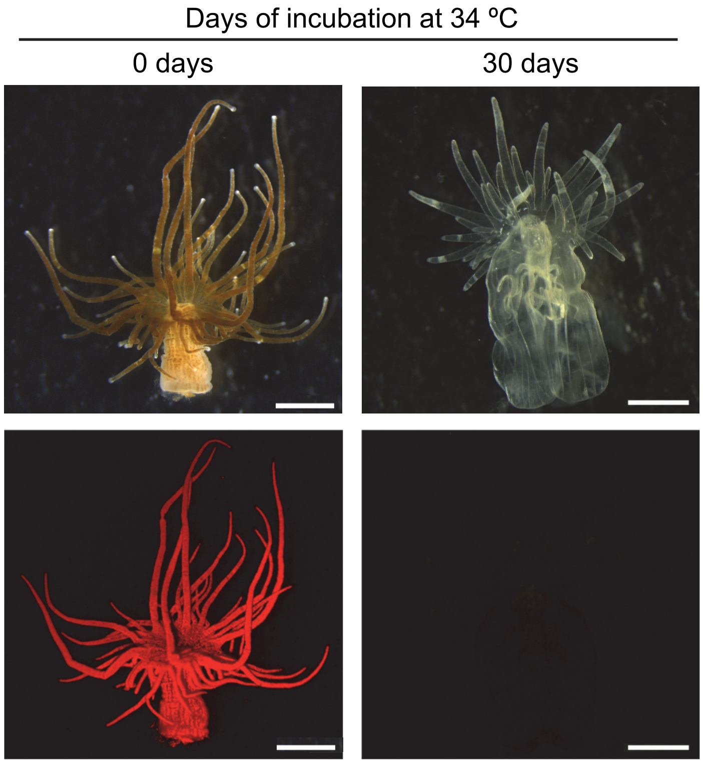

Supplementary Fig. 1 Photographs of Aiptasia with and without algae of Symbiodiniaceae. Aiptasia H2 polyps were incubated at 34 °C for 4 weeks to remove symbionts. Upper and lower photographs are light and fluorescence images, respectively. Red fluorescence represent chlorophyll fluorescence from algae. Scale is 1 mm.

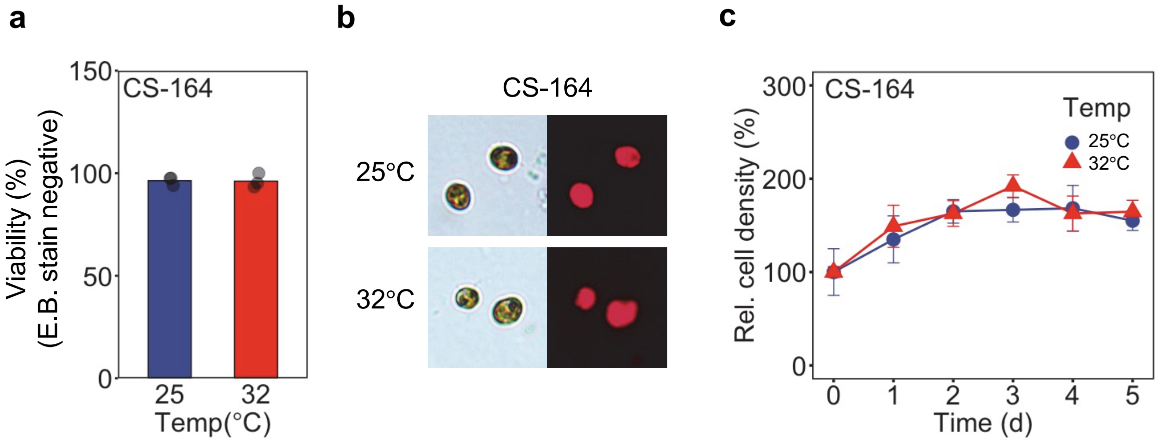

Supplementary Figure 2: Effect of high temperature stress on algal viability and cell number. Symbiodiniaceae CS-164 cells were incubated at 25 °C or 32 °C in dark. (a, b***)*** Algal viability was examined by Evans blue staining after the incubation for 3 days. (b) Photographs of CS-164 cells were taken after Evans blue staining in bright field (left) and in fluorescence (right) with a microscope under 32 X magnification. (c) Cell density was monitored during the temperature treatment for 5 days by counting cell number using a hemocytometer. Values are percentages of the initial 40,000 cell ml^{-1} (100%). Values are shown as mean±SE from three independent experiments.

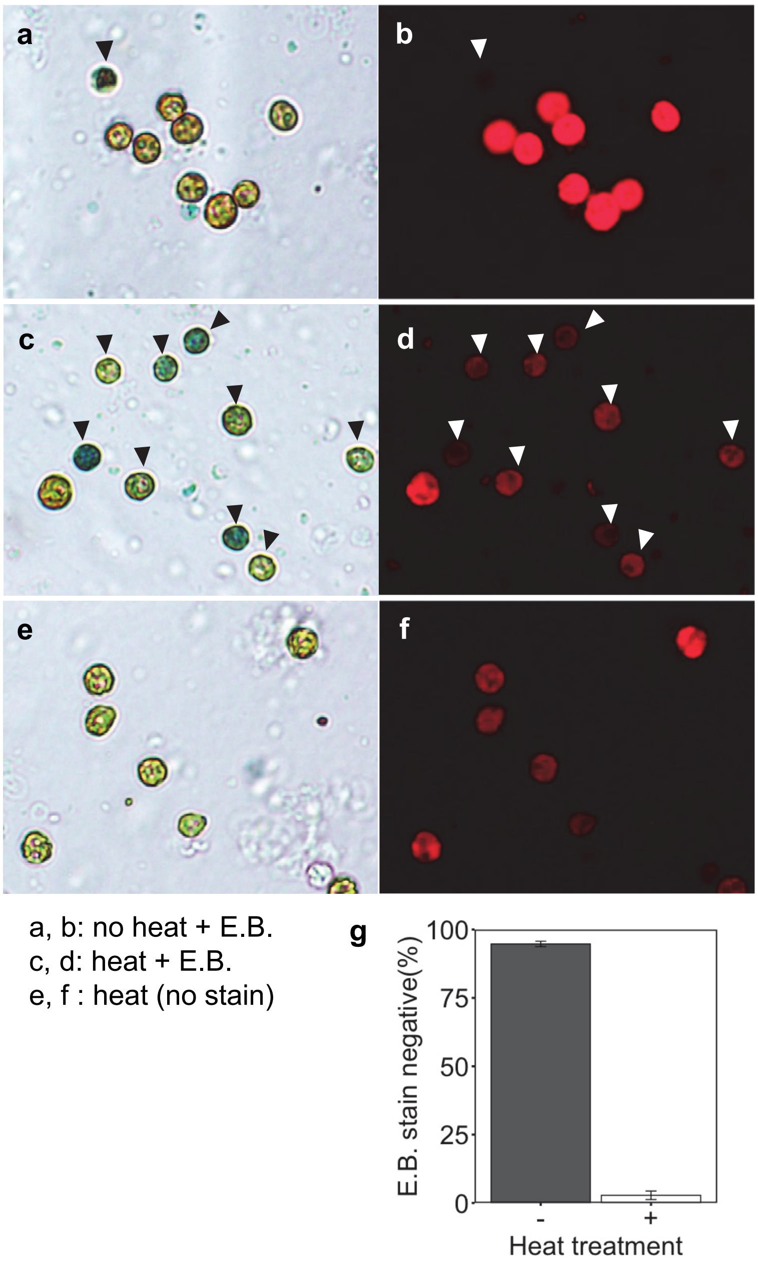

Supplementary Figure 3: Positive and negative control of Evans blue staining. Cultured algal cells of CS-164 were incubated at 80 °C in darkness for 1 h. Photographs of the cells were taken before (a, b) and after (c-f) the heat treatment, with (a-d) or without (e, f) Evans blue staining under 32 X magnification. Left side and right side are bright field images and fluorescence (WIG filter: Excitation 520~550nm band pass, Detection 580nm~) images, respectively. Evans blue stain-positive cells (dead cells) are indicated by black arrows in bright field images and white arrows in fluorescence images. (g) The ratios of stain-negative cells (living cells) were measured before and after the heat treatment.

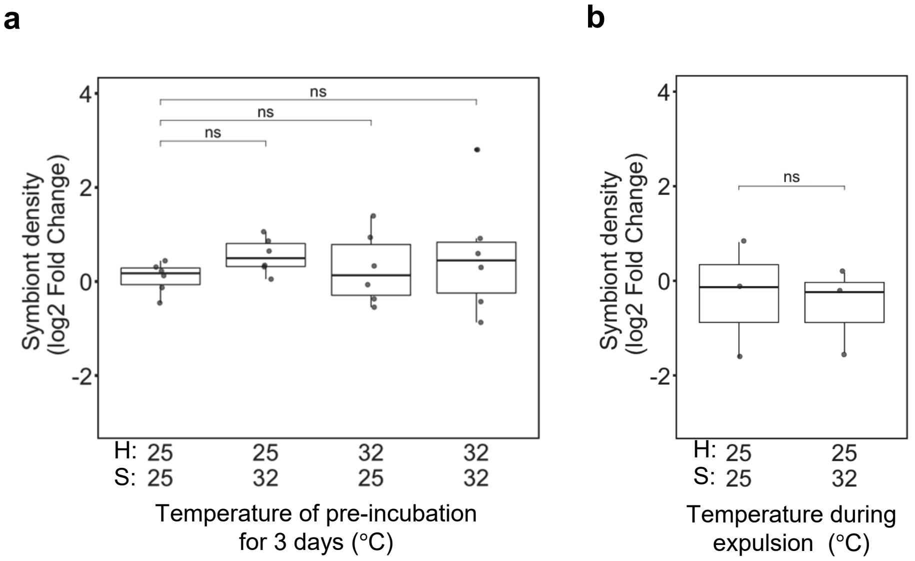

Supplementary Figure 4: Infectivity of *B. psygmophilum* CCMP2459 following exposure to elevated temperature. (a) The density of symbionts in tentacles was measured 3 days after culturing Aiptasia polyps (H) with symbiont cells (S) in four different treatments, as indicated below the panel and outlined in the text. (b) The density of symbionts was measured 3 days after culturing Aiptasia polyps with symbiotic algae in different treatments. In this experiment, symbiotic algae that had been expelled from Aiptasia polyps cultured at 25 ℃ or 32 ℃ for 3 days were used to infect Aiptasia. (a, b) Values are log2 fold changes with respect to the samples without any temperature treatment. Each point represents an independent experiment. ns, not significant (with $p>0.05$).
